# The Regulation of Artificial Intelligence in Digital Radiology in the Scientific Literature: A Narrative Review of Reviews

**DOI:** 10.3390/healthcare10101824

**Published:** 2022-09-21

**Authors:** Daniele Giansanti

**Affiliations:** Centro TISP, Istituto Superiore di Sanità, 00161 Roma, Italy; daniele.giansanti@iss.it

**Keywords:** regulation, artificial intelligence, digital radiology, medical devices

## Abstract

Today, there is growing interest in artificial intelligence (AI) in the field of digital radiology (DR). This is also due to the push that has been applied in this sector due to the pandemic. Many studies are devoted to the challenges of integration in the health domain. One of the most important challenges is that of regulations. This study conducted a narrative review of reviews on the international approach to the regulation of AI in DR. The design of the study was based on: (I) An overview on Scopus and Pubmed (II) A qualification and eligibility process based on a standardized checklist and a scoring system. The results have highlighted an international approach to the regulation of these systems classified as “software as medical devices (SaMD)” arranged into: ethical issues, international regulatory framework, and bottlenecks of the legal issues. Several recommendations emerge from the analysis. They are all based on fundamental pillars: (a) The need to overcome a differentiated approach between countries. (b) The need for greater transparency and publicity of information both for SaMDs as a whole and for the algorithms and test patterns. (c) The need for an interdisciplinary approach that avoids bias (including demographic) in algorithms and test data. (d) The need to reduce some limits/gaps of the scientific literature production that do not cover the international approach.

## 1. Introduction

### 1.1. Background

AI is increasingly bursting into many sectors of the *health domain*. One of the areas where the introduction of AI is increasingly being discussed is that of DR. Many healthcare activities seem to be able to benefit from AI. The benefits range from workload assistance, quality control, and clinical decision automation [1]. However, there are important steps that must be followed before the complete introduction of AI in a standardized way in clinical routine.

This review looks at the evolution of regulations with interest. The regulations are among the topics considered of great interest in the Special Issue “Assistive technologies, robotics and automated machines in the healthcare sector” [2] as they are connected to the standardization processes in the introduction of AI in the healthcare sector.

Recent experiences of use during the pandemic seem to have given an important push towards the affirmation and integration of AI in the health domain [3,4].

It is foreseeable that more and more studies dedicated to the transfer of evidence-based medicine to healthcare will be undertaken. Agreement tools used in the health domain will be useful and have been successfully used in many sectors. It is foreseeable that tools such as health technology assessment or comparative effectiveness research tools [5] will be increasingly used. Other methods, such as consensus conferences, may also have a successful application as in other sectors, such as that of care robots [6,7]. All this will allow us to obtain guidelines [8] on the use of AI in DR. *At the basis of all this, there will be the regulatory aspects that will be essential and preparatory in the processes of integration of consent through the tools listed above*.

### 1.2. Related Work

The Pubmed search had the following key [9]:

*Search: ((artificial intelligence[Title/Abstract]) AND (radiology[Title/Abstract])) AND (regulation) Sort by: Most Recent “artificial intelligence”[Title/Abstract] AND “radiology”[Title/Abstract] AND (“legislation and jurisprudence”[MeSH Subheading] OR (“legislation”[All Fields] AND “jurisprudence”[All Fields]) OR “legislation and jurisprudence”[All Fields] OR “regulations”[All Fields] OR “social control, formal”[MeSH Terms] OR (“social”[All Fields] AND “control”[All Fields] AND “formal”[All Fields]) OR “formal social control”[All Fields] OR “regulate”[All Fields] OR “regulates”[All Fields] OR “regulating”[All Fields] OR “regulation s”[All Fields] OR “regulative”[All Fields] OR “regulator”[All Fields] OR “regulator s”[All Fields] OR “regulators”[All Fields] OR “regulated”[All Fields] OR “regulation”[All Fields])*. It is noted that scientific research activity in this area is relatively recent with scientific works starting from 2018, demonstrating that it is a hot topic in relation to regulations and has only recently been addressed.

A total of 41 scientific papers emerge from the search, of which 16 are revisions demonstrating a still-limited scientific production. In the last 2 years (2021–2022), there are 16 contributions including 8 reviews. The contributions based on full scientific articles [10,11,12,13,14,15,16,17] dealt with various issues related to the regulations ranging from barriers for the radiologist [10], to the knowledge of this figure through surveys [13], to local experiences [12,13,14,15,16], to the implication of ethics [14], and the importance of federative activities [11]. Nair et al. [10] highlighted that there are barriers that radiologists should be aware of prior to implementing Artificial Intelligence in daily practice. Barriers include regulatory compliance, ethical issues, data privacy, cybersecurity, AI training bias, and safe integration of AI into routine practice. Castellanos et al. [11] faced the importance of the data federation in this field. Data federation offers a way to get data moving from multiple sources, providing advantages in healthcare systems where medical data is often hard to reach because of regulations or the lack of reliable solutions that can integrate on top of protocols such as FHIR, HL7, and DICOM, among others. They proposed an architectural solution that may provide the core capabilities to implement a data federation approach in a healthcare system to enable AI. Yy et al. [12] reported the regulatory approach proposed in their country, Korea. Eiroa et al. [13] focused on Spain and reported on an experience-based survey of radiologists, where regulation was among the topics. They concluded that, although there is a lack of knowledge about AI among Spanish radiologists, there is a will to explore such topics and a general belief that radiologists should be trained in these matters. Based on the results, a consensus is needed to change the current training curriculum to better prepare future radiologists. The contribution by Batle et al. [14] is a report of the ACR Data Sharing Work group, where the regulation issues are dedicated to Data Ethics of Privacy, Consent, and Anonymization. Allen et al. [15] focused on the algorithms, discussed why regulatory clearance alone may not be enough to ensure AI will be safe and effective in all radiological practices, and reviewed strategies and available resources for evaluation before clinical use and monitoring performance of AI models to ensure efficacy and patient safety. Kenny et al. [16] reported on the ethics and standards in the use of artificial intelligence in medicine based on the Royal Australian and New Zealand College of Radiologists. Harvey et al. [17] discussed the uncertain legal environment. In particular, they examined the nature, exposure, and theories of liability relevant to musculoskeletal radiologist practice with a particular focus on the negligence, vicarious liability, and product liability frameworks. 

### 1.3. Problems, Research Question, and Purpose of the Study

Now, scientific production in this area is rather scarce. As highlighted, scientific works have only recently been recorded in this area [9]. The topic of regulation is very important to allow the introduction of this technology in stable routine applications in the health domain. This regulation must address a wide range of issues that include many aspects, such as, to name a few, the development of algorithms, the certification of medical devices, ethical aspects, and workflows.

In this perspective, scientific studies based on reviews have the task of cataloguing and categorizing the various experiences in the international arena, to provide practical tools both to scholars and also to stakeholders. They can be useful, for example, as a starting point for addressing the production of guidelines, or as a support for consensus conferences. The main objective of this study is, therefore, to analyze how the regulatory aspects have been analyzed by these reviews, and aimed at answering the question “*How it is faced the regulation of artificial intelligence in Digital Radiology in scientific productions based on the reviews*” and make a map point on the issues where scholars have focused most. The main contribution of the study is to understand, through the analysis of these reviews, what the research trends are in this technological sector. The issues addressed by the reviews give an important idea, on the one hand, of the macro-research most faced by the scholars and, on the other hand, indirectly, on what the absent themes are in which it is necessary to insist the gaps be overcome.

## 2. Methods

The narrative review followed a standard narrative e checklist [18,19] and a qualification approach. The qualification methodology used an assessment based on a scoring scheme (with a score assigned to defined parameters) applied by two external experts not involved in the article, to include each reference. The followed checklist allowed to structure the design according to a standardized structure.

The experts were also required to add a comment to support their assessment.

Table 1 shows the parameters used in the scoring system. The score ranges from “1” (minimum-poor) up to ”5” (maximum-excellent). It was also checked and assessed for the exclusion/inclusion the conflicts of interest (declared, not declared, possible, etc.).

The work was included when all parameters had an assessment ≥3.

The Pubmed and Scopus Databases were analyzed and only publications in peer reviewed journals were considered.

The following search key was applied [20]:

Search: ((artificial intelligence [Title/Abstract]) AND (radiology [Title/Abstract])) AND (regulations) Filters: Review Sort by: Most Recent

*(“artificial intelligence”[Title/Abstract] AND “radiology”[Title/Abstract] AND (“legislation and jurisprudence”[MeSH Subheading] OR (“legislation”[All Fields] AND “jurisprudence”[All Fields]) OR “legislation and jurisprudence”[All Fields] OR “regulations”[All Fields] OR “social control, formal”[MeSH Terms] OR (“social”[All Fields] AND “control”[All Fields] AND “formal”[All Fields]) OR “formal social control”[All Fields] OR “regulate”[All Fields] OR “regulates”[All Fields] OR “regulating”[All Fields] OR “regulation s”[All Fields] OR “regulative”[All Fields] OR “regulator”[All Fields] OR “regulator s”[All Fields] OR “regulators”[All Fields] OR “regulated”[All Fields] OR “regulation”[All Fields])) AND (review[Filter]).*

## 3. Results

The search on Pubmed returned 16 papers [21,22,23,24,25,26,27,28,29,30,31,32,33,34,35,36] (Scopus returned 4 further works, which, being works at congresses in accordance with the qualification procedure, were excluded), all studies from 2018 to today.

The process of inclusion that was followed showed that:

- The number of reviews produced is very low, which denotes a low interest on the part of researchers in addressing the sector of AI regulations regarding digital radiology.
- No studies showed problems concerning the conflicts of interest.
- Two papers could be excluded [23,32], as they were not completely centered on the theme.

The study in [23] was excluded because radiology was treated marginally (as a neuroimaging tool) in the context of mental health care processes. It was considered from the analysis that the contribution relating to the regulatory aspects in this area was minimal.

The study in [32] was excluded as it was a sentiment analysis based on Twitter. The regulatory aspects were dealt with, but as a necessity that emerged from the tweets. Additionally, in this case it was considered that the contribution was minimal.

**Figure 1:**
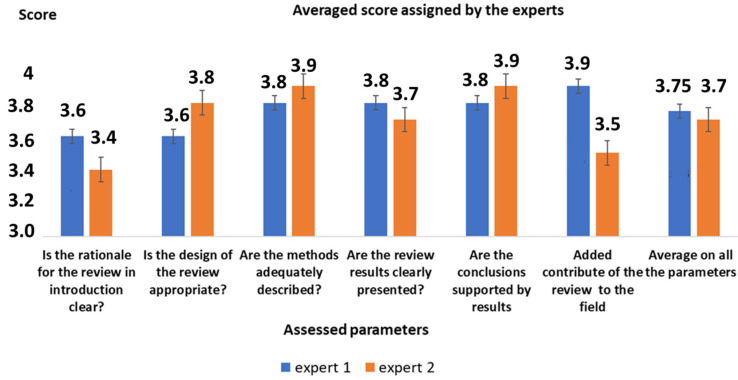
Output from the qualification process.

Figure 1 shows the outcome of the assessment procedure.

The analysis showed that the scientific literature has produced important and far-reaching reviews. Twelve reviews [21,25,26,27,28,29,30,31,33,34,35,36] out of fourteen were focused on radiology in general, two reviews were more specific, one review was focused on nuclear medicine [24], and one on imaging in gastrointestinal pathology [22]. As far as the regulatory aspects were concerned, some interesting issues emerge. Two papers addressed the issue from a more general point of view [21,28] by relating the regulations to the challenges. One focused exclusively on the ethical issues [26], other studies focused on the European [25,33] and American [25,29,33] situations, in some cases also making comparisons [25,33]. One study was related to the Canadian situation [30]. Two other studies offered new and important points of view on the regulatory aspects in this area; one focused on AI in DR, stressing the concept of *software as medical device* (SaMD) [27]; the other one dealt with the importance of providing certified datasets in training AI systems [31]. The three most recent reviews focused on some heterogeneous problems of AI regulation [34,35,36]. The review proposed in [34] faced the problems of regulating the workloads and the relevant implications. In addition, the hurdles of the legal issues to AI autonomy have been faced in [35], where the ambiguity of the legal management and the impact on all the actors were stressed. The imaging-based algorithms validated by the FDA were analyzed in [36] with the focus also on the emerging problems.

Artificial intelligence/machine learning (AI/ML) algorithms have been analyzed in [36]. 

What emerged from the search suggests an organized analysis based on the following topics: (a) Ethical issues in the scientific literature. (b) The regulatory framework in the scientific literature. (c) Bottlenecks of the legal issues.

### 3.1. The Ethical Issues in the Scientific Literature

Mudgal et al. [26] proposed a study entirely focused on the ethical issues. This review covered the different ethical issues of a harmless and justifiable deployment of AI in every part of the training, incorporation in the health domain, and regulation. According to the study, the managed data must: be appropriately evaluated, refined, purified, and managed in a centralized and non-dispersive way; be processed through methods that provide for anonymization, consent, and information to patients; represent all demographics. Transparency and security, according to the authors, must have central roles and all systems must follow authorization processes by the competent authorities. Ethical issues have been extensively addressed together with other issues in three other studies [21,24,30].

The review by Harvey et al. [21] considered both the power to redefine the practice of radiology and the nascent phase with the related largely untested aspects in the clinical space. The uncertainty of the legal-regulatory environment (also related to the ethical issues) according to the authors affected the introduction of AI. The study reported: (a) the challenges, tracing the various pathways toward approval by the FDA. (b) The future of government oversight. (c) The privacy issues. (d) The ethical dilemmas. (e) The practical considerations related to implementation in radiologist practice. The authors highlighted how, for nuclear medicine, careful considerations were needed for reliability, safety, non-maleficence, beneficence, justice and fairness, data privacy, data security, data confidentiality, minimization of bias, clearness, autonomy, and clarification ability. Jaremko et al. [30] produced a study for the Canadian Association of Radiologists focused both on regulatory and on ethical issues. The study reported a framework of the legal and ethical issues, with reference to the Canadian health domain. 

The review by Currie et al. [24] focused specifically on nuclear medicine. The study reported the strong opportunities of an “ethical AI” in terms of productivity and workflow, strengthening of the research, and clinical use. 

### 3.2. The Regulatory Framework in the Scientific Literature

Two studies among the overviewed ones faced the regulatory framework both for the European Union and for the US [25,33]. The study by Pesapane et al. [23] analyzed the regulation focusing on the legal framework of the Medical Devices (MDs) in the US and Europe. The study reported that European bodies were producing new regulations on cybersecurity, data protection, regulation of integrations, MDs, and in vitro MDs regulation. The FDA was controlling the scene in the US. The study emphasized that regulations for privacy protection, ethical use, and safety were required, and that AI applications must be considered MDs dedicated to diagnosis/detection. The approach in US and in Europe was different. Europe dedicated, for example, particular space to the cybersecurity, continuity of the services, and to the notification process. The regulations in the US were particularly focused on the processing and consent of the use of data and on the consent of the user/consumer. The review by Muehlematter [25] raised concerns on the approval processes for the AI-based MDs in Europe and the US. They identified 240 MDs approved in Europe and 222 in the US. They found that these numbers had increased considerably since 2015. Very few of these MDs were approved as high-risk MDs. The study concluded that the high number of approved MDs suggested ensuring severe regulation and a well-defined pathway for these MDs was lacking both in Europe and in the US. The authors recommended more transparency on the process of approval and management of these MDs and a complete, freely reachable database dedicated to these MDs.

Harvey et al. [29] focused on the US and remarked on the position in [25,33]. Furthermore, the authors analyzed the process of AI approval by the FDA. They reported that the FDA had proposed an innovative approach capable of minimizing the regulatory burden incumbent on the designers. The proposal of the FDA looked to the current good manufacturing practices and proposed to adopt a total product lifecycle method. Jaremko et al. [30] provided a study focused on Canada on both regulatory and ethical issues. Recommendations for patient data, algorithms, and practice were reported [30] with reference of the healthcare of this country.

The regulation of AI in radiology is increasingly being linked to the concept of MDs as shown by the studies reported in [25,33]. The review by Arora et al. [27] remarked that AI is recognized as a “Software as a Medical Device (SaMD)” and is looked at with this vision by the regulators. Authors turned around this concept, reporting that there is a strong interconnection between these AI systems with the Internet of Things, genetic data, and patient records. These important synergies must therefore be enclosed and considered when facing these MDs. The input data for testing a SaMD are also strategic. When it comes to SaMD, the problems are even more relevant. The study by Allen et al. [31] was in this direction. They reported that commercial algorithms were affected by gender, ethnic, and social bias. This shows important and dramatic implications in the design of algorithms in healthcare. They also reported that it is important to prevent biases in healthcare by means of a strong connection among the actors to assure robust and bias-free algorithms and datasets. They described a proposal solution at the ACR Data Science Institute. Here, a synergy among different institutions allowed the development of robust datasets and algorithms incorporating standards to mitigate biases. 

### 3.3. The Bottlenecks of the Legal Issues

The last three reviews published on these issues have focused specifically on bottlenecks in regulatory aspects [34,35,36] and represent mutually complementary and overall exhaustive contributions. Alexander et al. [34] focused on the impact of workload on the decision correctness. Many studies also focused on biology have shown that the decision speediness is inversely connected to the correctness. The study analyzed this issue in radiology also considering the impact of the AI. The authors concluded that regulating the workloads without a proper scientific approach could be more dangerous than the not regulating them. Mezrich [35] focused on the legal liability on the errors interconnected to AI use. The review identified several critical issues. One of these issues is the enforcement of the AI-based product liability law adopted in the US. The study identified ambiguities in the legal treatment of AI. The authors believe that this could profoundly affect the integration of AI into the health domain and the trust of all actors involved.

Ebrahimian et al. [36] focused on FDA-regulated AI algorithms. They reviewed 127 regulated software with the aim to classify the available and reported information. They recorded (when available) the number of studies included with other parameters, for example, the specificity, the receiver operating characteristic area under the curve, and the sensitivity. They reported an increasing number of MDs regulated by the FDA from the year 2008 up to 2021. Their review, very importantly, concluded that insufficient public data on the validation/testing datasets in different algorithms are not able to justify applications in healthcare as the generalization and/or the incidence of biases cannot be deduced.

## 4. Discussion

A deep analysis conducted on social media highlighted a growing attention on the integration of AI in DR within the health domain [32]. Surely, the COVID-19 pandemic that people are experiencing has also represented an important push in this direction [37,38,39] and given an important lesson for the future [40]. An improvement in equity of care is also expected from the integration of AI in DR into the health domain [41]. It is quite clear that integration into the health domain involves major challenges and processes of acceptance of consensus. 

In a previous study [42], the following was addressed: (a) the challenges in the growth and incorporation of AI in healthcare. (b) The acceptance and consensus on the integration of AI in healthcare. 

Among the many important challenges, the study highlighted the consolidation of an AI system as a MD and of the related regulatory framework, including ethics and emerging risks. Our overview conducted in Pubmed and Scopus focused precisely on this point. The number of reviews produced is very low, which denotes a low interest on the part of researchers in addressing the sector of AI regulations regarding digital radiology. This denotes a need for more efforts in this area by scholars.

The selected reviews focused on some specific aspects. They, therefore, gave a scientific contribution on certain aspects and problems. Some studies have focused on DR in general [21,25,26,27,28,29,30,31,33,34,35,36], while other contributions have investigated specific sectors [22,24]. Some studies faced this from the point of view of the challenges [21,28]. Other studies have focused on the European, American, and Canadian conditions [25,29,30,33]. An approach such as SaMD shines through in these studies. In fact, the need to address this issue considering that AI in DR is a SaMD appears evident in specific studies such as in [27]. The need to certify the datasets used in the SaMD is also considered indispensable [31]. Other studies have addressed specific aspects, such as ethics [16] and regulatory bottlenecks [34,35,36]. 

### 4.1. Added Value of the Review

The proposed overview aimed to give an added value by acting as a connector between all the specific issues addressed in this field to date, to give the scholar a vision of the facets that emerge in the research. Precisely all these facets that emerge together with the real fragmentation of regulatory approaches suggest to scholars from all over the world to meet and start consensus conferences to propose important recommendations that trigger both stakeholders and legislators. An indirect added value is represented by the discovery of the gaps/limitations emerging in the scientific literature production analyzed in the form of reviews.

### 4.2. Limitations of the Reviews

The subject of regulations is a subject that affects all nations and continents. The analysis of the reviews shows that scholars have focused essentially on European, US, and Canadian regulations. What one would expect from these reviews is that they address the problem with a greater angle and a more international and less local vision, i.e., not only the US, Europe, or Canada, which among other things are showing a mixed position. They should answer also to other important key questions, for example: How is the regulation in Asia and Africa? For example, from the National Medical Products Administration (NMPA), the Chinese agency for regulating drugs and medical devices (formerly the China Food and Drug Administration or CFDA)? From the Web, the position of the NMPA on these issues was found. The NMPA released three guidelines to regulate and support the rapid development in digital health also facing AI [43]:

Guideline for Artificial Intelligence in Medical Devices Registration. 

Guideline for Medical Device Software Registration.

Guideline for Medical Device Cybersecurity.

These guidelines represent an international harmonization effort. They are partly inspired by the position of the FDA and partly by the position of the IMDRF and are particularly focused on risk factors and product total life cycle management. In addition, additional reports and specific guidance documents are made available on AI and also with reference to DR [44]. It is evident that the international scientific literature should also give space through reviews to these initiatives that move towards processes of a uniformity of approach.

The regulatory impact relating to ethics is also addressed more from a local point of view. In the reviews, experiences and documents of a non-international nature are not shared, which could make a contribution, however, as regards a uniformity of approach.

In Europe, for example, it is possible to find several documents oriented towards this theme produced by the European community, such as a document dedicated to guidelines for a trustworthy AI and a document that addresses the impact [45] on public services [46].

In summary, there seems to be a fragmentation, or rather, a separation, between the production processes of scientific literature and what is present on public services in the governmental WEBs dedicated to this topic.

### 4.3. Comparison with the Recent Research Trends 

If we compare what emerges from the overview of reviews with the dedicated studies based on full scientific articles of 2021–2022 [10,11,12,13,14,15,16,17], it emerges that there is a coincidence of issues, such as the problem of barriers and critical issues of the issues [10] in relation to the introduction of AI in the health domain, the need for a federative approach [11], the importance of ethical aspects [16], and the interest in local situations as in [13,16].

However, what would have been expected from the overview of reviews is a role of cultural and scientific mediation between the various experiences. The analysis did not show this role.

There are studies, for example, on regulations in Korea [13], or studies on regulations in Australia and New Zeland [16], that have not been taken into consideration, preferring a discussion and analysis on the realities in Canada, Europe, and the USA.

The same goes for ethics, where other interesting studies have been found on the Australian continent that have not been resumed [16].

The need to overcome an analysis of regulatory realities with a patchy approach emerges strongly from this comparison with the studies based on full scientific articles [10,11,12,13,14,15,16,17] but also from the survey reported above of some government websites, where the regulatory approach is described [43,44] also with reference to ethics [45,46].

### 4.4. Limitations

Our study, based on a narrative review, has several limitations. The review considered papers written in English. The reviews in different languages were not considered. PubMed and Scopus databases, and only peer reviewed papers available here, were considered in the review. A possible enlargement of the overview could consider databases including conference papers, preprint sources, and governmental WEBs. The theme faced in this study is very wide and included several sub-themes (e.g., regulations in MDs, regulations in the production of the datasets). Future enlargements could comprise reviews directed in the identified sub-themes.

## 5. Conclusions

The development of a solid regulatory framework is indispensable today in the field of AI in DR. This study conducted a narrative overview on reviews to make a map point in this field and explore trends and gaps in the scientific literature production. 

It is possible to detect some achievements in brief.

### 5.1. Achievements in Brief

The *first achievement* is that the overview reported a patchy approach limited to some countries showing a not uniform approach.

The *second achievement* is the need of a more transparent approval process, from an international and contemporary point of view, and the need for open database sources for Medical Devices, algorithms, and datasets.

The *third achievement* is the need of a process of harmonization at the international level of the approach.

The *fourth achievement* is for the ethics, where the need of avoiding demographic bias in the datasets and algorithms was highlighted.

The *fifth achievement* consists in the detected bottlenecks, such as, for example, the difficulty in identifying the workloads for the radiologists and the insufficient transparency in the validation of the datasets, not justifying the use in some applications.

### 5.2. Conclusions in Detail

In conclusion, the overview highlighted a low production (only 16 reviews) in this field and the need for more efforts of the scientists. The review has identified three important areas of intervention: *ethics issues, international regulatory framework, and bottlenecks in the regulatory development*. Regarding ethics, the areas of intervention have been identified, which, in addition to the traditional ones of the use of technologies in healthcare, include new ones due to the specificity of AI connected to the production of the algorithms, datasets, and avoiding the bias in them. Studies on regulations, before all, highlighted that those emerging regulatory approaches were not uniform and differed in the case of the US, Europe, Canada, or other countries. Different approaches were used to address emerging issues, such as cybersecurity in MDs. The studies have revealed several critical issues, and, in particular, the need to assure a well-defined and rigorous pathway for the approval and the maintenance of the AI-based MDs. Among the recommendations for these issues, more transparency on the approval and post-approval process and the design of a full, openly reachable database dedicated to these MDs was proposed. Bottlenecks were also identified with particular reference to the workload, showing that regulating it without a scientific principle may be more dangerous than no regulation at all. Reference was also made to the insufficient public data on the validation/testing of the datasets in different algorithms not justifying applications in healthcare as the generalization and/or the incidence of biases cannot be deduced. Most importantly, *limitations and gaps* emerge in these reviews. It emerged that they limit themselves to considering regulatory experiences that do not cover the international approach. For example, some significant experiences are not considered, such as those of the NMPA, which may have a role of legislative mediation between multiple positions. Also with regard to ethical aspects, it would be advisable to better share important documentary production experiences, such as those available in Europe.

## Figures and Tables

**Table 1:** Parameters used for the qualification (standardized table).

PARAMETER ASSESSED
Is the rationale for the review in the introduction clear?
Is the design of the review appropriate?
Are the methods described clearly?
Are the results presented clearly?
Are the conclusions based and justified by results

## Data Availability

Not applicable.

## Acronyms

AI	Artificial Intelligence
DR	Digital radiology
MD	Medical Device
SaMD	Software as Medical Device
FDA	Food and Drug administration
ML	Machine Learning
ACR	American College of Radiology
NMPD	National Medical Products Administration
IMDRF	International Medical Device Regulators Forum
FHIR	Fast Healthcare Interoperability Resource
DICOM	Digital Imaging and COmmunications in Medicine
HL7	Health Level seven
